# Noninvasive estimation of tumour viability in a xenograft model of human neuroblastoma with proton magnetic resonance spectroscopy (^1^H MRS)

**DOI:** 10.1038/sj.bjc.6600704

**Published:** 2003-02-10

**Authors:** M Lindskog, P Kogner, F Ponthan, P Schweinhardt, B Sandstedt, T Heiden, G Helms, C Spenger

**Affiliations:** 1 Childhood Cancer Research Unit, Department of Woman and Child Health, Karolinska Institutet, Karolinska Hospital, S-171 76 Stockholm, Sweden; 2 MR-Centre, Department of Clinical Neuroscience and Department of Neuroscience, Karolinska Institutet S-171 76 Stockholm, Sweden; 3 Research Group Tumor Genetics and Molecular Genetics, Institute of Medical Genetics, Charite, Humboldt University of Berlin, Germany

**Keywords:** neuroblastoma, xenograft, spectroscopy, MRI, angiogenesis, therapy

## Abstract

The aim of the study was to evaluate proton magnetic resonance spectroscopy (^1^H MRS) for noninvasive biological characterisation of neuroblastoma xenografts *in vivo*. For designing the experiments, human neuroblastoma xenografts growing subcutaneously in nude rats were analysed *in vivo* with ^1^H MRS and magnetic resonance imaging at 4.7 T. The effects of spontaneous tumour growth and antiangiogenesis treatment, respectively, on spectral characteristics were evaluated. The spectroscopic findings were compared to tumour morphology, proliferation and viable tumour tissue fraction. The results showed that signals from choline (Cho)-containing compounds and mobile lipids (MLs) dominated the spectra. The individual ML/Cho ratios for both treated and untreated tumours were positively correlated with tumour volume (*P*<0.05). There was an inverse correlation between the ML/Cho ratio and the viable tumour fraction (*r*=−0.86, *P*<0.001). Higher ML/Cho ratios concomitant with pronounced histological changes were seen in spectra from tumours treated with the antiangiogenic drug TNP-470, compared to untreated control tumours (*P*<0.05). In conclusion, the ML/Cho ratio obtained *in vivo* by ^1^H MRS enabled accurate assessment of the viable tumour fraction in a human neuroblastoma xenograft model. ^1^H MRS also revealed early metabolic effects of antiangiogenesis treatment. ^1^H MRS could prove useful as a tool to monitor experimental therapy in preclinical models of neuroblastoma, and possibly also in children.

Treatment with cytotoxic and cytostatic agents in addition to surgery is the cornerstone of clinical therapy for childhood solid tumours. Alteration in tumour size, assessed by magnetic resonance imaging (MRI), computed tomography (CT) or ultrasound, is currently the basis for the evaluation of treatment response (Berdon *et al*, 1992; Hiorns and Owens, 2001). This evaluation is usually performed after weeks or months of treatment, during which the child may suffer from severe side effects and not always benefit from the therapy.

The development of sensitive methods for repeated and noninvasive measurement of tumour biological activity during treatment needs to be attempted in order to optimise and individualise the treatment of childhood solid tumours. MRI is currently the method of choice for radiological evaluation of many childhood tumours and is increasingly used in clinical routine (Finelli *et al*, 2000; Shapeero and Vanel, 2000; Siegel *et al*, 2002). It is easily accessible in most paediatric cancer centres, considered safe and generally well tolerated.

The use of proton magnetic resonance spectroscopy (^1^H MRS) can complement MRI in useful ways. ^1^H MRS allows sampling of detailed biochemical information from normal tissues or pathological processes and has proved valuable for the characterisation of a wide range of malignant disorders (Negendank, 1992). ^1^H MRS shares many similarities with conventional MRI (for basic principles of ^1^H MRS and MRI, see previously published reviews (Howe *et al*, 1993; de Certaines, 1996)). Using ^1^H MRS, metabolic information is obtained by suppressing the water contribution to the received echo followed by frequency analysis of the remaining signal. Individual molecules may thus be identified based on their inherent individual frequency fingerprints (chemical shift from water). A molecular configuration that enables a high level of proton mobility is a prerequisite for detection with ^1^H MRS. Accessible metabolites include a large number of intracellular compounds such as phospholipids and polypeptides.

Metabolites with chemical shifts between 0.9 and 1.4 ppm are frequently observed in ^1^H MRS spectra of human tumours (Guidoni *et al*, 1987; Le Moyec *et al*, 1992; Negendank *et al*, 1996). These signals principally correspond to fatty acyl chains with methylene (CH_2_) and methyl (CH_3_) groups, and are commonly designated mobile lipids (MLs). Several reports indicate a possible correlation between the amount of MLs and tumour cell death (Kuesel *et al*, 1994, 1996). Choline-(Cho) containing compounds (chemical shift=3.2 ppm) are abundant during the synthesis and degradation of cellular membranes. As Cho-containing compounds are characteristically elevated in many malignant tumours their relative signal intensity has been suggested as an indicator of grade of malignancy (Usenius *et al*, 1994; Tedeschi *et al*, 1997).

Neuroblastoma, an embryonal tumour of the sympathetic nervous system, is the most common extracranial solid tumour in children. The clinical course is highly heterogeneous ranging from spontaneous involution (as sometimes seen in infants) to rapid tumour dissemination and a dismal prognosis in the majority of older children. Metastases to bone or bone marrow or the finding of certain specific genetic aberrations (e.g. MYCN amplification, 1p deletion, 17q gain) (Seeger *et al*, 1985; Caron *et al*, 1996; Bown *et al*, 1999) in the tumour cells are major adverse prognostic factors. For these patients long-term survival is seldom achieved despite intensive multimodal treatment (Matthay *et al*, 1999). The availability of repeated noninvasive assessment of treatment response could contribute to improved outcome for children with high-risk neuroblastoma by early evaluation of novel therapy and application of individualised treatment.

In the present work, we employed a xenograft model of human neuroblastoma in an attempt to evaluate the biological relevance of metabolites detected by ^1^H MRS *in vivo*. In particular, we investigated Cho and MLs. We were able to show a significant inverse correlation between the ML/Cho ratio and the viable tumour fraction. Compared to untreated controls, tumours treated with an angiogenesis inhibitor showed higher ML/Cho ratios concomitant with marked histological changes at a time point when tumour volumes were still unaffected by treatment.

## MATERIALS AND METHODS

### Neuroblastoma cells

The human neuroblastoma cell line SH-SY5Y (was kindly provided by Dr June Biedler, New York) (Biedler *et al*, 1973). Cells were cultured at 37°C in a humidified 95% air/5% CO_2_ atmosphere. Eagle's minimum essential medium was supplemented with 10% foetal bovine serum, L-glutamine 2 mM, penicillin G 100 IU ml^−1^ and streptomycin 100 *μ*g ml^−1^ (Gibco BRL, Paisley, Scotland, UK). The medium was changed twice weekly and confluent cultures were subcultivated after treatment with 0.5 g l^−1^ trypsin and 0.2 g l^−1^ EDTA (Gibco BRL). Cultures were free from mycoplasma as verified by DNA staining. For xenotransplantation, a single-cell suspension, approximately 10^8^ cells ml^−1^, was prepared in culture medium supplemented with L-glutamine (2 mM). The viability and exact cell concentration were assessed by trypan blue dye exclusion using a haematocytometer.

### Animal model

A total of 20 male nude rats (HsdHan: RNU-rnu, Harlan, Netherlands) at the age of 5–6 weeks were used for the establishment of xenografts as previously described (Nilsson *et al*, 1993). In short, animals were anaesthetised with Hypnorm (Janssen Pharmaceutical, Beerse, Belgium) and 2×10^7^ cells suspended in 0.2 ml medium were injected into each hind limb. A 23-gauge cannula was used to deposit the suspension subcutaneously (s.c.). Attention was paid so as not to pierce the muscle fascia or lose cells by leakage from the injection site.

### Ethics

The experiments described herein were approved by the Regional Ethics Committee for Animal Research. They also meet the ethical standards required by the United Kingdom Co-ordinating Committee on Cancer Research (UKCCCR, 1998) Guidelines.

### Quantification of tumour growth

Tumour take was defined as the observation of a palpable and measurable nodule in the area of cell injection. Tumours were measured with a digital calliper every other day and tumour volume was calculated by length×width^2^×0.44 (Wassberg *et al*, 1997). Tumours were allowed to reach a maximum length of 25 mm. Tumours were detectable in 18 out of 20 rats within 10–20 days after cell inoculation and grew with a doubling time of 3–5 days. Animals without tumour take were excluded from the study. TNP-470-treated animals were followed for 10 days and the tumour volumes at day 10 were 1.15±0.56 ml (mean±s.d.), *n*=8, range 0.74–2.4 ml. Control animals were killed at different time points (days 4–21) with final tumour volumes of 3.4±2.9 ml (mean±s.d.), *n*=9, range 0.4–8.6 ml. In this group, the two largest tumours were followed up to a size of approximately 25 mm, which represented days 19 and 21 from tumour take, respectively.

### Experimental treatment

Animals were randomly assigned to either control (*n*=10) or treatment group (*n*=8). Animals in the treatment group received the antiangiogenic compound TNP-470 (kindly provided by Dr Rolf Christofferson, Uppsala) 10 mg kg^−1^ s.c. every other day for 10 days (Wassberg *et al*, 1997). Control animals received no treatment.

### Magnetic resonance imaging and, ^1^H MRS

MRI examinations were performed using a 4.7 T magnet with horizontal bore (Bruker Biospec Avance 47/40, Bruker, Karlsruhe, Germany). The magnet was equipped with a 12 cm inner diameter self-shielded gradient system (max. gradient strength 200 mT m^−1^). Tumours were investigated using a circular surface coil with a planar circular detection area of 3 cm. The surface coil was fitted into a customised Plexiglas rig placed in the centre of the gradient system. In order to facilitate the positioning, the rat was placed on a separate rig in side position. Animals were anaesthetised with isofluorane and respiratory activity was monitored continuously. Body temperature was registered and maintained at 37±0.5°C with a heated air stream. The manufacturer's implementation of RARE sequence was used for anatomical imaging, with the following parameters: T1-weighted images: repetition time (TR)=535 ms, echo time (TE)=10 ms; T2-weighted images: TR=2500 ms, TE=115 ms; proton density-weighted images: TR=2500 ms, TE=35 ms.

For MRS, the STEAM sequence, which uses three lobe sinc pulses, was employed for selection of volumes of interest (VOIs) and additional spoiling gradients. VOIs were carefully inscribed within the xenografts to avoid spectral contamination from adjacent muscle or fatty tissue. The VOI was chosen to include a maximal volume of the tumour, and thus varied with tumour size between approximately 0.2 and 6 cm^3^. Care was taken so that the included volumes were as representative as possible for the whole tumour appearance as viewed in MRI. In a few cases, shimming difficulties lead to the choice of somewhat smaller volumes. The VOI approximately represented 50–80% of the total tumour volume.

After suppression of the water signal, acquisitions were averaged (64–128) until a signal-to-noise ratio sufficient for spectral evaluation was reached. A long TR (6 s) was employed in order to achieve full relaxation between scans and to avoid different degrees of saturation because of differences in T1 and spatial variation of the excitation flip angle. The TE was varied to differentiate between the trimethyl singlet of Cho-containing compounds (chemical shift=3.22 ppm), multiple of macromolecules/lipid resonances (ML) and weakly coupled lactate (Lac, 1.33 ppm). The first spectrum was recorded at 135 ms, where the slowly relaxing Cho is preserved and Lac is inverted. If a residual signal of fast relaxing lipids was present, the measurement was repeated at TE=270 ms to improve the relaxation differentiation. Finally, a spectrum at short TE=20 ms was recorded showing all metabolites with negligible T2 weighting. In addition, spectra from skeletal muscle and subcutaneous fat were recorded to identify corresponding contamination. This was found to be negligible. The metabolic levels were estimated by peak area integration after appropriate baseline correction. For calculation of the ML/Cho ratio, the methylene lipid peak appearing at 1.2–1.4 ppm (TE=20) was employed.

In the control group, MRI/MRS was performed at time points between days 4 and 21 from tumour take in order to study the influence of tumour volume on spectroscopic characteristics. In the treatment group MRI/MRS was performed after completion of therapy (day 10±1). In both groups, there was a sufficient variation of tumour size such that spectroscopic measurements could be correlated to the volumes. On the day of MR-examination, the tumour volume was measured as described.

### Morphology and assessment of tumour viability 

Animals were killed by an overdose of pentobarbital immediately after the MRS examination. Tumours were excised, fixed in 10% formalin and embedded in paraffin. Sections (5 *μ*m) were obtained from different parts of each tumour and processed for histology. Tumour cell proliferation was assessed using a rabbit monoclonal antibody raised against the tumour proliferation marker Ki-67 (Ki-67, rabbit anti-human, A047. Dakopatts, Stockholm, Sweden). A Ki-67 labelling index (LI) was calculated from 1500 nuclei at a magnification of ×400 in three randomly chosen viable areas. The terminal deoxynucleotidyl transferase (TUNEL) reactivity assay (ApopTag, S7100, Intergen, NY, USA) was employed for quantification of apoptosis (Gavrieli *et al*, 1992). Haematoxylin–eosin (HE) staining was used for routine morphology. The Scion Image computer software package (Scion Corp.) was employed for calculating necrotic/apoptotic areas and viable tumour areas, respectively. The overall tumour viability was estimated by calculating the surface areas of viable regions in three different randomly chosen longitudinal tumour sections (at least one section being close to the centre of the tumour). The viable tumour fraction was expressed as the mean value of these measurements in percent for each individual tumour. For cells to be classified as viable, findings of well-defined borders and intact nuclei were obligate. An investigator blinded to treatment status and MRS results performed the morphological evaluation.

### Statistical analysis

Statistical analysis was performed using appropriate software (SYSTAT, Inc., Evanston, IL, USA). Parameters were correlated to tumour volume or viable tumour fraction in univariate regression models and the Pearson correlation factors were calculated. For comparison between different groups, *t*-values were calculated as previously described (Evtouchenko *et al*, 1996) and compared to the critical value, t* of the Student's distribution at 1−*q*^(0.975)^.

## RESULTS

### MRI and ^1^H MRS

**Figure 1:**
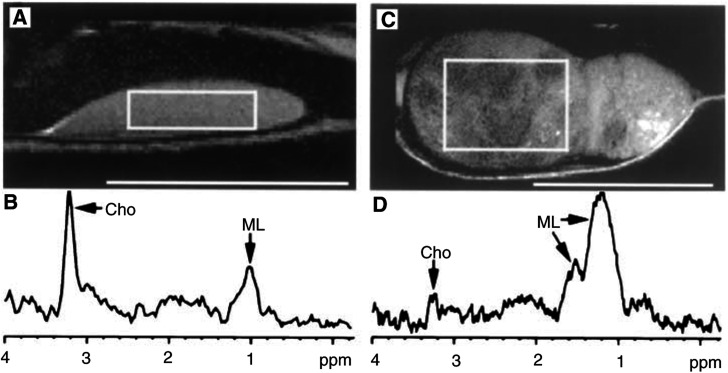
(**A,C**) Proton density-weighted MR images of two representative xenografts two (**A**) and three (**C**) weeks after cell inoculation, respectively. Scale bars=10 mm. (**B**) ^1^H MRS spectrum from tumour in **A**. The selected VOI is indicated by a rectangle. Cho-containing compounds dominate the spectrum. ML signals are also seen. TE=20 ms, 128 averages. (**D**) ^1^H MRS spectrum from large tumour seen in **C**. The VOI (rectangle in **C**) includes tumour tissue with variable signal intensity. There is a weak Cho signal while MLs dominate. TE=20 ms, 128 averages. **B** and **D** are not shown to scale.

Tumours <1.5 ml generally appeared homogeneous but occasionally contained foci with low signal intensity on T2 images. Such low signal areas were seen more often in larger tumours. All tumours >2 ml had a heterogeneous appearance, containing both hyperintense and hypointense areas (Figure 1

).

**Figure 2:**
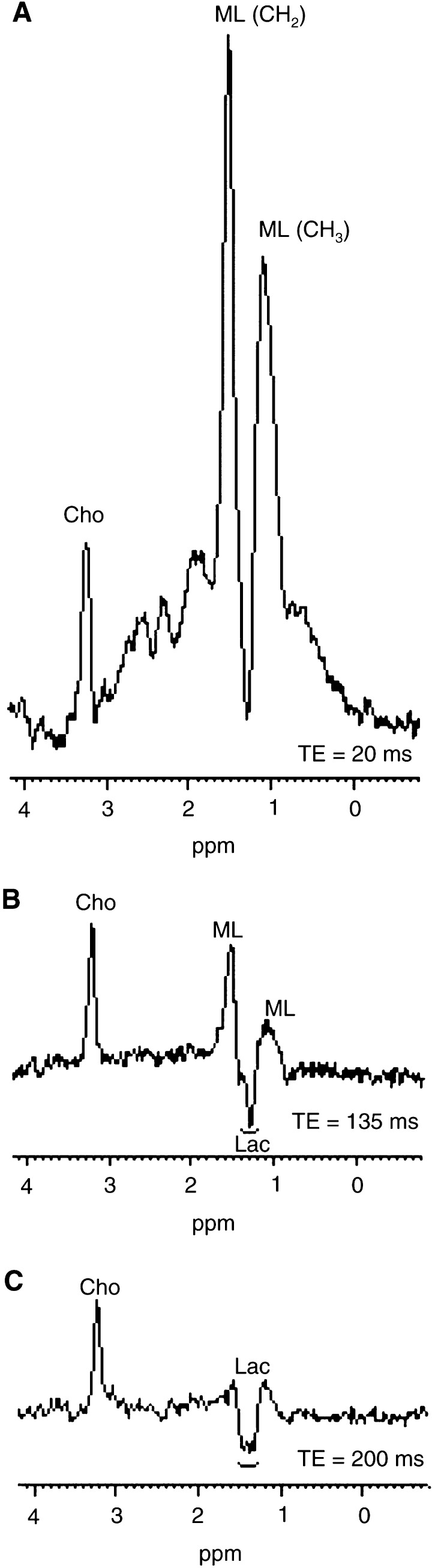
(**A–C**) TE dependence of spectra obtained from a largely necrotic xenograft. (**A**): TE=20 ms. MLs dominate over a large range of chemical shifts (0–3 ppm) and obscure the Lac doublet resonance at 1.33 ppm. (**B**) TE=135 ms. MLs have been reduced by faster T_2_ relaxation to the level of Cho. Interference of Lac (inverse at 135 ms) with residual lipid resonances is clearly seen. (**C**) TE=200 ms. Relaxation differentiation on long T_2_ metabolites (Cho, Lac). The fast relaxing ML signal has vanished exhibiting the lactate doublet (antiphase dispersion lines).

In MR spectroscopy, short TE spectra (TE=20 ms) were dominated by signals from Cho at 3.2 ppm and ML signals, the latter contributing to resonances at 0.7–0.9 ppm and 1.2–1.5 ppm, respectively (Figure 1 and Figure 2

).

**Figure 3:**
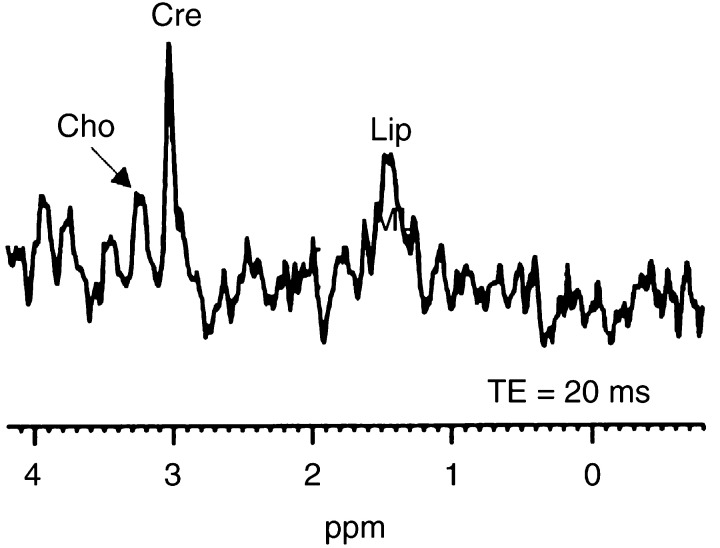
Control spectra from skeletal muscle in a nude rat. The resonance of creatine/phosphocreatine (Cre) at 3.0 ppm was not detectable in neuroblastomas. Cho=choline, Lip=lipids. TE=20 ms, 64 averages.

Spectra from small xenografts (*V*<1.5 ml) typically had intense Cho signals together with moderate ML levels (Figure 1b). A small peak at 3.0 ppm, compatible with creatine/phosphocreatine, seemed to be present in most tumours but could not be unequivocally separated from Cho and was therefore not analysed further. The putative neuronal marker NAA (*N*-acetyl-aspartate) (Urenjak *et al*, 1992) with a chemical shift of 2.0 ppm was not distinguishable at short TEs where lipid signals overlapped with this spectral region. Neither intermediate (TE=135 ms) nor long (TE=172 ms) TEs (where lipid signals are fully relaxed) could support any presence of NAA in the xenografts (Figure 2). Spectra obtained from large tumours (*V*>2 ml) generally displayed a ML peak of high intensity at 1.2–1.4 ppm, a variable lipid peak at 0.9 ppm and a less prominent Cho signal (Figure 1 and Figure 2). Control spectra obtained from skeletal muscle showed a prominent creatine peak (only weakly present in tumour spectra) and a modest Cho signal (Figure 3

).

### Morphology and tumour viability

**Figure 4:**
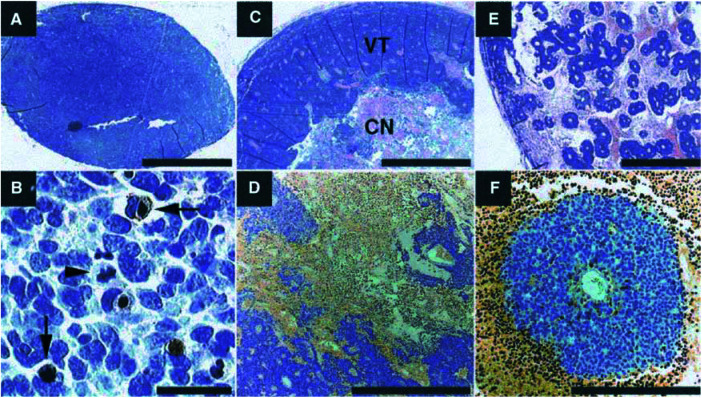
(**A**) Overview of small, homogeneous tumour (HE). (**B**) Same tumour as in **A** (TUNEL and HE). Apoptotic cells brown (arrows). Cell undergoing mitosis is marked with arrowhead. (**C**) Large untreated tumour with extensive central necrosis (CN) and a peripheral viable tumour tissue (VT) (HE); (**D**) Same tumour as in **C** (TUNEL). (**E**) Overview of a tumour treated with TNP-470 (HE). Note multiple small round perivascular cuffs of tumour cells surrounded by extensive necrosis and haemorrhage. (**F**) Same tumour as in **E** (TUNEL). Scale bar=2 mm applies to **A** and **C**. Scale bar in **B**=25 *μ*m, in **D**=500 *μ*m, in **E**=1 mm, in **F**=250 *μ*m, respectively.

Small tumours (*V*<1.5 ml) generally showed a dense and mostly viable appearance on macroscopic examination while larger tumours were mainly necrotic and haemorrhagic. These macroscopic findings were corroborated to microscopic analyses (Figure 4A–F

). Microscopically viable tumour regions had the characteristic appearance of poorly differentiated neuroblastoma (Figure 4A,B) (Shimada *et al*, 1999b). In nonviable areas, HE staining demonstrated a diffuse type of cell death with necrosis, apoptosis, haemorrhage and infiltrating inflammatory cells (Figure 4C). Nonviable areas were strongly TUNEL positive (Figure 4D). The proliferation index in viable tumour areas showed little variation (LI=30±4%) between tumours, irrespective of treatment or overall tumour viability (data not shown).

### Angiostatic treatment

**Figure 5:**
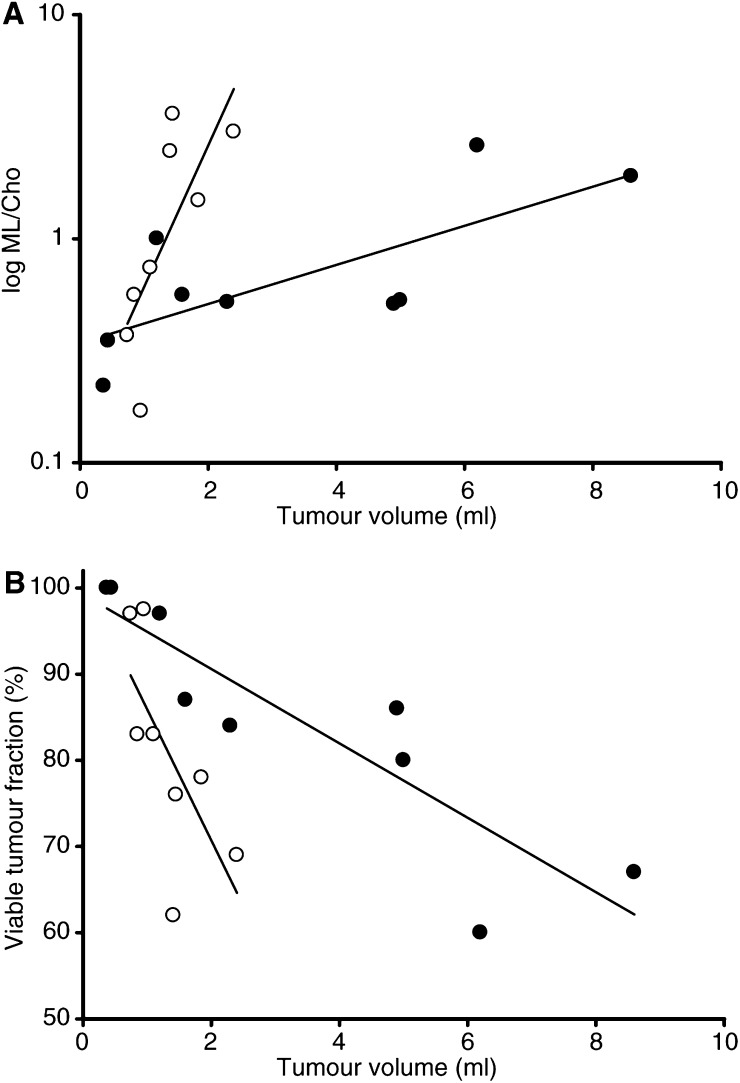
(**A**) The ML/Cho ratio (here shown as log value) correlated significantly to tumour volume for treated (open circles, *P*<0.05) and untreated (solid circles, *P*<0.05) tumours. The regressions of control and treatment groups were significantly different (*P*<0.05). (**B**) In untreated tumours, viable fraction correlated inversely with tumour volume (*r*=−0.88, *P*<0.05). A comparable trend was also seen for treated tumours, though with steeper slope (*r*=−0.68, *P*=0.058).

Histological examination of tumours from animals treated with TNP-470 demonstrated a characteristic appearance with perivascular cuffs of viable tumour cells embedded in nonviable areas (Figure 4E,F). This was not observed in untreated tumours. When compared to tumour volume, a tendency for lower viable fraction in the treatment group was observed (Figure 5B

). TNP-470-treated tumours did not differ from untreated tumours of corresponding size on appearance on MRI. Comparison of tumour volumes between treatment and control groups on day 10 from tumour take did not show any significant difference between the two groups (data not shown).

### Metabolic findings in relation to tumour characteristics

**Figure 6:**
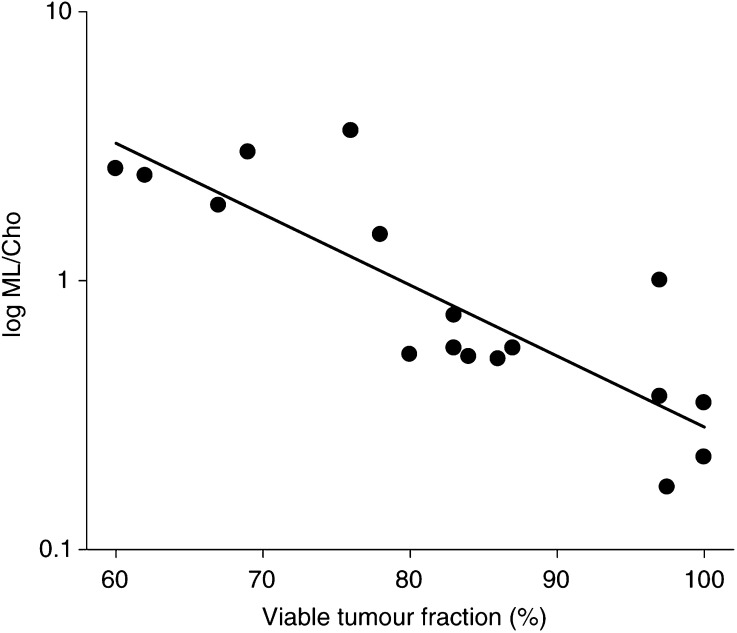
Significant inverse correlation between ML/Cho-ratio and viable tumour fraction in human neuroblastoma xenografts (*r*=−0.86, *P*<0.001) (data pooled from TNP and control groups).

The ML/Cho ratio was significantly correlated with tumour volume for both treated and untreated tumours (*r*=0.75, *P*<0.05 and *r*=0.73, *P*<0.05, respectively) (Figure 5A). In the treatment group ML/Cho ratios increased faster with tumour volume than in untreated animals (*P*<0.05). Viable tumour fraction was negatively correlated to tumour volume in untreated animals (*r*=0.88, *P*<0.001) (Figure 5B). In treated animals there was a tendency towards a similar correlation (*P*=0.058). Comparison with histological findings revealed a significant inverse correlation between the ML/Cho ratio and the viable tumour cell fraction (*r*=−0.86, *P*<0.001) (Figure 6

). This relation was valid for both treated and untreated tumours. There was no correlation between cell proliferation (Ki-67 index) and ML/Cho ratio (data not shown).

## DISCUSSION

Noninvasive methods that allow repeated evaluation of tumour status and response to therapy could improve efforts to individualise treatment of cancer patients. Proton magnetic resonance spectroscopy is emerging as a highly useful tool for this purpose (Hakumaki *et al*, 2002; Kauppinen, 2002). In the present work, we evaluated the relevance of MLs and Cho-containing compounds detected *in vivo* by ^1^H MRS in a xenograft model of human neuroblastoma. We found that the ML (methylene)/Cho ratio constitutes a noninvasive measure of the viable tumour fraction in this tumour model.

Only a few previous studies employing ^1^H MRS in neuroblastoma have been reported. Intense creatine and Cho signals were found in ^1^H MRS spectra from perchloric extracts of neuroblastoma cells. (Florian *et al*, 1995). When neuroblastoma cells were implanted in rat brain, a prominent Cho peak but no creatine was detected *in vivo* with ^1^H MRS (Gyngell *et al*, 1994). MLs were not analysed in these previous studies because of the extraction procedure and TE employed, respectively.

Conventional MRI may detect necrosis in xenograft tumours and was suggested as a marker of treatment response (Jakobsen *et al*, 1995). However, necrosis as a possible *in vivo* marker of successful treatment may be too unspecific as it frequently occurs spontaneously in rapidly growing solid tumours. Furthermore, in some tumour types, a high necrotic fraction was found to correlate to worse outcome (Bjornsson *et al*, 1993; Pierallini *et al*, 1998). In neuroblastomas, focal necrosis and haemorrhage are common (Shimada *et al*, 1999a). We observed that neuroblastoma xenografts developed extensive areas of necrosis and haemorrhage during spontaneous growth (Figure 4). Therefore, we suggest that assessment of the viable tumour fraction would likely be a more accurate indicator of treatment response in neuroblastoma than necrosis alone. Several investigators have demonstrated an accumulation of MLs in areas of apoptosis/necrosis (Kuesel *et al*, 1996; Negendank and Sauter, 1996). Furthermore, *in vitro* studies have shown that the ratio of the methylene (CH_2_) lipid resonance (at 1.3 ppm) to the methyl (CH_3_) resonance (at 0.9 ppm) is correlated to the percentage of apoptotic lymphoblasts after doxorubicin treatment (Blankenberg *et al*, 1997). This finding was recently confirmed using a human cervical carcinoma HeLa cell system (Bezabeh *et al*, 2001). In the present study, we found strong ML signals in large, highly necrotic tumours. However, although less prominent, MLs were also detected in a subset of viable non-necrotic tumours. Thus, in the present work MLs did not *per se* indicate cell death. Consequently, events other than apoptosis or necrosis may also be responsible for the lipid resonances at 0.7–1.5 ppm. This is in accordance with previous reports demonstrating the presence of MLs in viable tumour areas (Kuesel *et al*, 1994; Negendank and Sauter, 1996). Recently, we performed ^1^H spectroscopic investigations of a panel of human neuroblastoma cell lines in culture and found prominent ML signals in viable cells (Lindskog *et al*, unpublished observation). This observation is in line with the finding of ML droplets inside growth-compromised, but viable, glioma cells (Barba *et al*, 1999). Furthermore, in activated lymphocytes the amount of MRS-visible lipids correlates with the proportion of cells in late S and G phase (Veale *et al*, 1997). To clarify the biological significance of lipid resonances, further studies aiming at linking factors such as cell cycle distribution and tumour microenvironment (oxygenation, nutrient supply and pH) to the dynamics of ^1^H MRS visible lipids may be required.

Phosphocholine (PCho) is one of the major contributors to the Cho resonance observed in ^1^H MRS spectra (Bhakoo *et al*, 1996; Ackerstaff *et al*, 2001). The PCho concentration was shown to correlate with the number of cells in S phase in a xenograft model of breast cancer (Smith *et al*, 1991). In human astrocytoma the Cho/crea time ratio correlates with proliferation index (Ki-67), while the NAA/Cho ratio is inversely correlated with Ki-67 (Tamiya *et al*, 2000). Thus, it was suggested that the Cho signal reflects cellular proliferation in these tumours. In our present study, neither creatine nor NAA signals were unequivocally accessible for spectroscopic analysis, nor were we able to show any correlation between ML/Cho ratio and proliferative activity (Ki-67). The antiangiogenic compound TNP-470 is an inhibitor of endothelial cells (Castronovo and Belotti, 1996) and has been shown to reduce the growth of neuroblastoma xenografts, causing increased necrosis and apoptosis (Wassberg *et al*, 1997; Katzenstein *et al*, 1999). In experimental studies of antiangiogenic drugs, tumour volume measurements have generally been employed for *in vivo* assessment of treatment efficacy. Figure 5A shows that ML/Cho ratios of both treated and untreated tumours correlate significantly with tumour volume. Further, the stronger increase in ML/Cho ratio as a function of tumour volume in treated tumours suggests a decreased viability. This is verified in Figure 5B. Based on these observations, and since measurement of tumour volume may include both viable and necrotic areas, we suggest that spectroscopic information could usefully complement volume measurements for the evaluation of experimental treatment in neuroblastoma.

In the present work, we evaluated the possibility of noninvasive detection of early effects in response to TNP-470 in experimental neuroblastomas, using ^1^H MRS. After 10 days of treatment, there was no significant difference in tumour volume between the two groups. We could, however, detect higher ML/Cho ratios in relation to tumour volume in treated tumours (Figure 5A). Concomitantly, marked histological changes were seen in TNP-470-treated tumours (Figure 4E,F) similar to those previously reported (Wassberg *et al*, 1997). Taken together, our observations suggest that the higher ML/Cho ratios detected after short-term TNP-470 treatment indicate early antitumour effects as reflected in the histological appearance. The partial overlap between the treatment and the control group in tumours smaller than 1 ml and with ML/Cho ratios below 0.5 (Figure 5A) may be explained by the fact that these tumours (two treated and two untreated) were highly viable. This suggests the existence of two ‘poor responders’ in the treatment group. It needs to be further explored as to whether ^1^H MRS could serve as a noninvasive method to monitor efficacy in longitudinal studies with antiangiogenic drugs.

The SH-SY5Y xenograft model employed in this study is a well-characterized and highly reproducible *in vivo* model of poorly differentiated human neuroblastoma (Nilsson *et al*, 1993; Ponthan *et al*, 2001). Caution should however be taken not to generalise our findings to all neuroblastoma tumours since this is a highly heterogeneous disease. To further address a potential role of ^1^H MRS for the monitoring of neuroblastoma biology and, importantly, for validating the efficacy of novel antitumour therapies, additional studies are needed. Performing repeated *in vivo* ^1^H MRS examinations, we recently observed increases in ML/Cho ratios in neuroblastoma xenografts during chemotherapy (Lindskog *et al*, unpublished observation). In the present paper, we provide evidence that the ML/Cho ratio inversely correlates to tumour viability. Hence, we suggest that ^1^H MRS could be a tool for monitoring the treatment of neuroblastoma in children by repeated examinations during the course of therapy.

In conclusion, we have shown that the viable tumour fraction can be accurately estimated *in vivo* with ^1^H MRS in a model of human neuroblastoma. Our findings also suggest that the ML/Cho ratio may be valuable as a noninvasive surrogate marker to detect response to experimental therapy in neuroblastoma.
